# Path Optimization for Cluster Order Picking in Warehouse Robotics Using Hybrid Symbolic Control and Bio-Inspired Metaheuristic Approaches

**DOI:** 10.3390/biomimetics10100657

**Published:** 2025-10-01

**Authors:** Mete Özbaltan, Serkan Çaşka, Merve Yıldırım, Cihat Şeker, Faruk Emre Aysal, Hazal Su Bıçakcı Yeşilkaya, Murat Demir, Emrah Kuzu

**Affiliations:** 1 Department of Electrical and Electronics Engineering, Faculty of Engineering and Architecture, İzmir Bakırçay University, 35665 İzmir, Türkiye; cihat.seker@bakircay.edu.tr (C.Ş.); hazalsu.bicakci@bakircay.edu.tr (H.S.B.Y.); murat.demir@bakircay.edu.tr (M.D.); 2 Department of Mechanical Engineering, Hasan Ferdi Turgutlu Technology Faculty, Manisa Celal Bayar University, 45400 Manisa, Türkiye; serkan.caska@cbu.edu.tr; 3 Department of Software Engineering, Faculty of Engineering, Karadeniz Technical University, 61080 Trabzon, Türkiye; merveyildirim@ktu.edu.tr; 4 Department of Mechanical Engineering, Faculty of Engineering, Giresun University, 28200 Giresun, Türkiye; faruk.aysal@giresun.edu.tr; 5 Department of Computer Technologies, Salihli Vocational School, Manisa Celal Bayar University, 45300 Manisa, Türkiye; emrah.kuzu@cbu.edu.tr

**Keywords:** path optimization, cluster order picking, warehouse robotics, symbolic discrete controller synthesis, metaheuristic approaches

## Abstract

In this study, we propose an architectural model for path optimization in cluster order picking within warehouse robotics, utilizing a hybrid approach that combines symbolic control and metaheuristic techniques. Among the optimization strategies, we incorporate bio-inspired metaheuristic algorithms such as the Walrus Optimization Algorithm (WOA), Puma Optimization Algorithm (POA), and Flying Foxes Algorithm (FFA), which are grounded in behavioral models observed in nature. We consider large-scale warehouse robotic systems, partitioned into clusters. To manage shared resources between clusters, the set of clusters is first formulated as a symbolic control design task within a discrete synthesis framework. Subsequently, the desired control goals are integrated into the model, encoded using parallel synchronous dataflow languages; the resulting controller, derived using our safety-focused and optimization-based synthesis approach, serves as the manager for the cluster. Safety objectives address the rigid system behaviors, while optimization objectives focus on minimizing the traveled path of the warehouse robots through the constructed cost function. The metaheuristic algorithms contribute at this stage, drawing inspiration from real-world animal behaviors, such as walruses’ cooperative movement and foraging, pumas’ territorial hunting strategies, and flying foxes’ echolocation-based navigation. These nature-inspired processes allow for effective solution space exploration and contribute to improving the quality of cluster-level path optimization. Our hybrid approach, integrating symbolic control and metaheuristic techniques, demonstrates significantly higher performance advantage over existing solutions, with experimental data verifying the practical effectiveness of our approach. Our proposed algorithm achieves up to 3.01% shorter intra-cluster paths compared to the metaheuristic algorithms, with an average improvement of 1.2%. For the entire warehouse, it provides up to 2.05% shorter paths on average, and even in the worst case, outperforms competing metaheuristic methods by 0.28%, demonstrating its consistent effectiveness in path optimization.

## 1. Introduction

The Order Picking Problem (OPP) is widely considered to be a highly critical and costly processes in warehouse logistics. It is essential to efficiently select products from the warehouse and prepare them for shipment in a timely manner, as well as to ensure the cost-effective storage of products that need to be stored. The rapid growth of e-commerce and the diversification of customer demands have made it imperative to conduct warehouse operations in a faster and error-free manner. Warehouse robots automate this process, enhancing both operational efficiency and reducing human errors. The optimal performance of these increasingly utilized warehouse robots in such processes is of paramount importance for achieving economic and safety objectives.

Inspired by biological systems, many recent optimization strategies have been developed based on biomimetic principles. In this study, we adopt bio-inspired metaheuristic algorithms that emulate natural phenomena such as pumas’ territorial hunting patterns, walruses’ cooperative foraging behavior, and flying foxes’ echolocation-based navigation. These biological mechanisms serve as the foundation for robust and adaptive optimization processes in our proposed framework.

In existing research, numerous studies have been conducted on order picking optimization for warehouse robots. Ref. [1] examines the use of mobile robots in the warehouse order picking process. A new vehicle routing problem is defined, and a model has been constructed to enhance the operational efficiency of the robots. Ref. [2] investigates the optimization of human–robot collaborative order picking in robot-assisted material handling systems. The optimization of pod selection and scheduling and manual picking operations is addressed using adaptive large neighborhood search. Ref. [3] examines the scheduling of human–robot coordinated order picking processes in intelligent part-to-picker systems. The goal is to reduce the overall picking time by employing a stochastic dynamic programming model.

Based on our extensive literature review, it has been observed that the most significant problem in warehouse robotic order picking is the coordination of robots and the vehicle routing problem. Our research idea is directly related to addressing this problem within the scope of this study.

When examining the literature on cluster order picking optimization in warehouse robots, it is generally addressed using either classical control methods or heuristic approaches. However, classical control methods often lead to high timing complexity, while heuristic methods may fail to ensure the reliability of safety-critical systems. In this study, we aim to combine symbolic discrete controller synthesis with metaheuristic approaches, targeting both superior performance and low timing complexity.

Discrete Controller Synthesis (DCS) was initially modeled by [4] as a theory of language. Subsequently, the regulation of systems governed by discrete events has been addressed using various modeling formalisms [5]. Work based on controller synthesis by [6] is one of the pioneering studies in this field. However, these studies do not address infinite reactive systems. In [7]’s work, discrete controller synthesis was extended to infinite systems. Ref. [8], on the other hand, further extended the symbolic discrete controller synthesis approach to input–output systems. On the other hand, studies related to optimization focus on minimizing the total cost over a time window through a cost function [9].

In the study by [10], symbolic control was used to manage traffic, aiming to ensure the safe flow of traffic and reduce congestion through the use of a controller. Ref. [11] compared the altitude problem in unmanned aerial vehicles using metaheuristic optimization algorithms. In [12], a modeling framework was presented, showing that robotic systems can be systematically treated as a symbolic discrete control synthesis problem.

When examining the studies in the literature, it is observed that approaches to path optimization for order picking in warehouse robots primarily involve metaheuristic optimization algorithms. However, metaheuristic optimization techniques used in isolation do not provide formal correctness. On the other hand, classical control techniques used in these systems often suffer from high computational complexity and are not sufficiently effective in terms of performance. To address this gap in the literature, we propose a hybrid approach. Our proposed hybrid approach not only scales well in terms of computational complexity but also guarantees formal correctness with high-performance results.

Contributions: We propose a modeling framework for path optimization in cluster order picking within warehouse robotics, utilizing a hybrid approach that combines symbolic control and metaheuristic techniques. Our approach broadly consists of the following components:

- Cluster Modeling: Uncontrolled system behaviors and desired control objectives are systematically modeled at the cluster level within the ReaX environment using parallel synchronous languages;
- Metaheuristic Optimization: Within each cluster, the models are trained using various metaheuristic optimization algorithms;
- Implementation: We validate our approach through experimental evaluation in the MATLAB (version R2021a, MathWorks, Natick, MA, USA) environment and report the findings.

The rest of the paper is structured as follows. The following subsection presents a detailed review of the relevant literature. A summary of the technical components in cluster order picking for warehouse robotics is given in Section 2. Section 3 presents our modeling framework that combines hybrid symbolic control and metaheuristic path optimization for cluster order picking in warehouse robots. The validation of our results is provided through the experimental evaluations in Section 4. In the last section, the conclusion and future work are presented in Section 5.

### 1.1. Related Work

#### 1.1.1. Cluster Order Picking and Warehouse Robots

To model in-warehouse order picking routes, Rymarczyk et al. [13] used SPRP algorithms and achieved a fourfold reduction in travel distance through wave-based order grouping. In Ref. [14], an optimization model incorporating collaborative robots to reduce knee load and minimize travel time and bending motions was developed. This model shows a 14.5–27.9% reduction in total time in real-world simulations. Their method improved both robot efficiency, by grouping related products into the same pods, and human picker performance, supporting sustainable warehouse management. In Ref. [15], an integer programming model based on historical order data was utilized to reduce shelf movements, thereby enhancing operational efficiency. In the context of smart hospital pharmacies, Yuan et al. [16] introduced a two-stage SSCA strategy for human–robot collaboration, significantly cutting down both machine visits and order preparation times. Building on AI-driven solutions, Kalkha et al. [17] presented an intelligent SLA method that leverages product demand patterns. Their method, which employs the AHC algorithm for high-turnover product grouping, reduced the order fulfillment time by up to 69%.

#### 1.1.2. Control Methods in Robotics and Optimization Studies

Ref. [18] offered an in-depth overview of formal controller synthesis techniques categorized by system models and safety requirements. Ref. [19] integrated a reachset-compatible model with formal synthesis techniques to ensure that modeled safety guarantees are transferred to actual robots. An LPV controller design for Cartesian robots via convex optimization was proposed in ref. [20]. This design achieved superior performance and significantly reduced engineering costs through automation.

Ref. [21] proposed a data-driven control framework which guides stochastic systems to optimal steady-state behavior utilizing only historical data. Theoretical progress in gradient-based policy optimization has been comprehensively reviewed by Hu et al. [22], with optimization structure, convergence properties, and robustness in control-related applications. In ref. [23], a control synthesis framework tailored for discrete-time nonlinear systems, employing the barrier method was introduced. This approach was analyzed, with convergence formally established and quadratic constraints thoroughly examined. In ref. [24], a closed-loop structure was introduced for discrete linear systems based on a shifted optimization interval and a quadratic performance criterion, presenting a method that eliminates the need for real-time optimization.

#### 1.1.3. Metaheuristic Optimization Algorithms in Robotics and Path Planing Studies

In mobile robotics, metaheuristic algorithms have seen increasing use in path-planning and Inverse Kinematics (IKs). Xu et al. [25] offered a new classification framework for such algorithms, while ref. [26] compared several methods to solve the IK problem in redundant robotic arms, utilising the UR5 and SIA20D platforms. Moreover, ref. [27] developed a hybrid control technique integrated with improved GWO for autonomous flying robots. This technique improves tracking performance and the convergence speed.

Ref. [28] utilized a HHO-GWO hybrid algorithm for path-planning and tracking in complex terrain, achieving outperformed results compared with conventional algorithms (e.g., PSO and GWO). In a different review study [29] discussing research on UAV path planning between 2018 and 2022, the dominance of hybrid algorithms and the increasing interest in multi-UAV and real-time 3D planning scenarios were emphasized. Finally, ref. [30] demonstrated the impact of the DGBCO algorithm for UAV deployment in disaster scenarios, delivering the best cost and time performance under harsh conditions.

## 2. Overview of Technical Aspects in Cluster Order Picking for Warehouse Robotics

Robotics is an interdisciplinary field at the intersection of engineering, control systems, and artificial intelligence. Modern robotic systems have the capabilities of perception, decision-making, and movement. Thus, they can perform complex tasks autonomously. Thanks to the development of sensor technologies, embedded systems, and machine learning algorithms, robots are used effectively not only in the manufacturing sector; they are also used effectively in areas such as agriculture, logistics, exploration missions, and healthcare. Humanoid robots and multirobot systems constitute important research topics in terms of human–robot collaboration. The field of robotics has an increasing importance in the literature because it develops innovative solutions for both theoretical knowledge production and application.

The operational success and adaptability of a robotic system is largely proportional to the competence of the implemented control algorithms. In scenarios where multiple robot platforms serve a common purpose, such as logistics and warehouse automation, elements such as path planning, task assignment, and collision avoidance become critical requirements. The ability of robots to adapt to dynamic environmental changes, overcome unforeseen obstacles, and use the most efficient routes are factors that directly affect the overall output of the system. Hybrid control strategies supported by artificial intelligence and machine learning techniques are proposed for complex and uncertain environments where traditional rigid control approaches are inadequate. These approaches enable robots to make smarter and more flexible decisions by learning from their environment, increase their level of autonomy, and offer the potential to successfully complete more complex tasks.

### 2.1. Robotic Arm Manipulators

**Figure 1:**
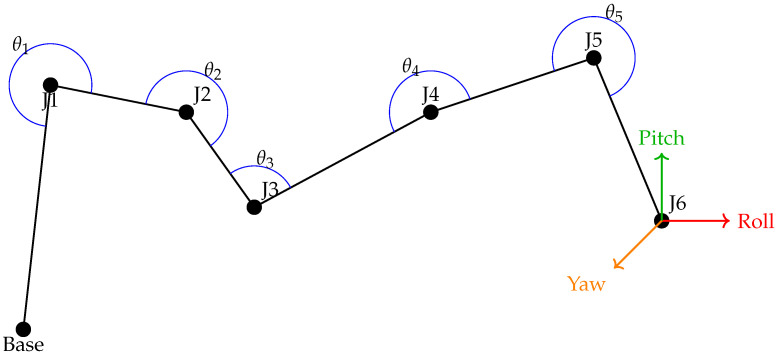
Joint configuration and angles of a 6-DOF robot arm.

Robotic arm manipulators are one of the fundamental building blocks of industrial automation and are commonly used in missions that require high mobility and precision. These systems usually have multiple degrees of freedom and can execute complex three-dimensional movements due to their jointed structures, as shown in Figure 1. Robotic manipulators are controlled using multidimensional mathematical approaches such as forward kinematics, inverse kinematics, dynamic modeling, and trajectory planning. In addition to control algorithms, robotic arms can be made safer in tasks that require human–robot interaction by integrating visual perception and force feedback systems. Today, the use of robotic arm manipulators is increasing in various application areas such as surgical robots, rehabilitation robots, and space robots, as well as in production lines.

Position control, speed control, and force control are commonly used control methods in robotic arm manipulators. Position control ensures that the tip of the robotic arm manipulator reaches a certain point precisely. In force control, the force applied or perceived by the robotic arm manipulator while interacting with its environment is regulated. These control loops are usually fed with data from internal sensors such as joint angle sensors, accelerometers, and torque sensors. In addition, in some cases, external sensors (e.g., cameras, LiDAR, tactile sensors) are integrated to increase the interaction of the robotic arm with the working environment. This sensor data allows the robot to perceive its environment, recognize objects, and perform dynamic tasks. The combination of AI and machine learning techniques in advanced robotic systems allows robotic arm manipulators to move more adaptively and autonomously, thus expanding their application areas.

### 2.2. Unmanned Ground Vehicles

Unmanned ground vehicles are mobile platforms armed with various sensing systems and decision-making algorithms, capable of operating remotely or autonomously. These types of vehicles are commonly utilized in mission areas such as disaster response, demining, border security, and military reconnaissance, where human intervention is risky and difficult. Unmanned ground vehicles can operate effectively with the integration of many subsystems, such as environmental perception, route planning, obstacle recognition, and motion control. Intelligent technologies like machine learning, artificial intelligence, and sensor fusion have seen a growing integration into unmanned ground vehicles to increase their resilience to environmental uncertainties and enhance their mission flexibility. However, multi-vehicle coordination and swarm-based control approaches have become widely used in the defense industry and various civilian applications.

The position and orientation of a wheeled unmanned ground vehicle can be modeled at a basic level by means of the kinematic differential equations presented below, which describe the heading angle and motion of the wheeled unmanned ground vehicle over time:

(1) $$\left\{\begin{array}{c} \dot{x}\left(t\right)=v\left(t\right)\cos\theta \left(t\right)\\ \dot{y}\left(t\right)=v\left(t\right)\sin\theta \left(t\right)\\ \dot{\theta }\left(t\right)=\omega \left(t\right) \end{array}\right.$$

where,

- x(t),y(t): the vehicle’s coordinates in the global reference frame, representing its position over time,
- θ(t): the heading angle, indicating the vehicle’s orientation with respect to the global reference frame,
- v(t): the linear velocity, defining the speed at which the vehicle moves along its heading direction,
- ω(t): the angular velocity, describing the rate of change of the heading angle θ(t) over time.

The interaction of unmanned ground vehicles with the environment, including path planning and the control of behaviors such as altitude and attitude, and decision making is crucial. Various sensor data, such as LiDAR, radar, and camera, are obtained. These data are then processed to ensure that vehicles reach their destinations safely and quickly. Dynamic obstacle avoidance strategies are implemented. A strong energy management system and a reliable communication infrastructure must be established for autonomous operations. These components directly affect the long-term and uninterrupted mission execution capability of unmanned ground vehicles. Technical components must be continuously developed in order for unmanned ground vehicles to be used successfully in more complex and uncertain scenarios.

### 2.3. Warehouse Robots and Path Optimization

Warehouse robots are designed to increase the efficiency and veracity of logistics operations. They can be autonomous, semi-autonomous, mobile, and fixed. These systems reduce human intervention in shelf placement, product picking, packaging, load handling, and inventory management processes and reduce labor costs. Warehouse robots developed by large-scale logistics companies such as Amazon, Alibaba, and Ocado are integrated with real-time data analysis and cloud-based control systems. They are one of the concrete examples of Industry 4.0. At this point, warehouse robots not only increase the level of automation but also play a strategic role in the transition to flexible production and distribution models.

Increasing efficiency by bringing multiple orders together in the same picking process in warehouse operations is called cluster order picking. In this method, robots simultaneously collect orders from different customers. Thus, total movement time and energy consumption are minimized. Advanced route planning algorithms and order clustering optimizations are used to reduce traffic density in the warehouse and shorten the pick-up time. In addition, these robots, which can work integrated with real-time data processing and barcode/RFID systems, significantly increase order accuracy. Cluster order picking, especially in warehouse systems with large volumes and a wide variety of products, both reduces operational costs and increases customer satisfaction.

Path optimization is the determination of the route that provides the shortest way to reach a specific destination for one or more agents using mathematical or algorithmic methods. It is at the intersection of many disciplines, such as robotics, transportation, logistics, intelligent transportation systems, and network design. It is generally based on minimizing a number of limiting criteria such as distance, time, energy consumption, or cost. Path optimization problems are structurally in the NP-hard class. Therefore, heuristic or metaheuristic algorithms are often preferred to exact solutions.

Mathematically, path optimization can be expressed as minimizing the total travel distance over an ordered sequence of waypoints, as shown in Equation (2):

(2) $$\min_{P}\sum_{i=1}^{n-1}d\left(p_{i},p_{i+1}\right)$$

where $P=\{p_{1},p_{2},\ldots ,p_{n}\}$ is the ordered set of points (e.g., picking locations) and $d(p_{i},p_{i+1})$ is the distance between consecutive points $p_{i}$ and $p_{i+1}$. The set $P=\{p_{1},p_{2},\ldots ,p_{n}\}$ represents a sequence of picking points that is ordered according to certain optimization criteria, such as minimizing the total Euclidean distance, reducing travel time, or avoiding congestion, typically using metaheuristic methods.

### 2.4. Metaheuristic Algorithms

Metaheuristic optimization algorithms have been developed for large, complex, and nonlinear problems with a solution space. These algorithms are not specific to a particular problem and can be easily applied to different optimization scenarios with parameter changes. They also offer effective solutions to difficulties such as constrained optimization, multiobjective optimization, and dynamic environments. Today, new-generation algorithms are being developed by combining artificial intelligence techniques and hybrid metaheuristic structures. Thus, solution quality and computational efficiency are increased. In this respect, metaheuristic algorithms are a powerful toolkit for optimization problems both in practical applications and in theoretical research.

**Figure 2:**
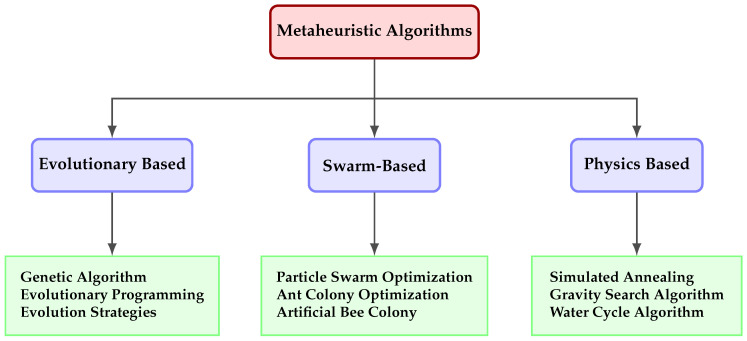
Classification of metaheuristic algorithms.

Metaheuristic algorithms have a wide range of applications, and Figure 2 illustrates their classification. They provide successful solutions to complex optimization problems encountered in many disciplines such as medicine, engineering, finance, and data science. They are effectively used in route optimization, facility layout, scheduling problems, and pattern recognition in large datasets. In recent years, hybrid approaches have been obtained by combining different metaheuristic algorithms. It has been observed that the integration of these approaches and adaptive mechanisms increases the performance of the algorithms. In the future, it is envisaged to develop metaheuristic algorithms that can work in harmony with big data and real-time systems, require less parameter adjustment, and have autonomous learning capabilities.

## 3. Hybrid Symbolic Control and Metaheuristic Optimization for Cluster Order Picking

**Figure 3:**
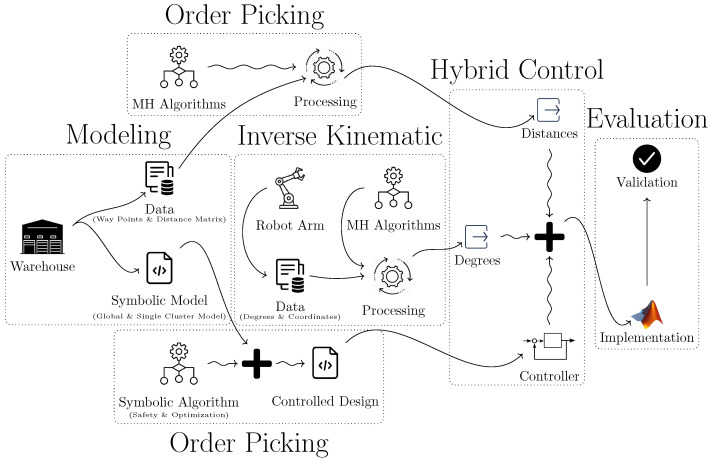
Schematic representation of the proposed approach.

In this study, we propose a path optimization approach for warehouse robotics in the context of cluster order picking. Our method is a hybrid solution that combines metaheuristic techniques with symbolic control to efficiently address the path planning problem. A schematic representation of the proposed approach is presented in Figure 3.

The warehouse considered in this study is large-scale and composed of multiple clusters. Within each warehouse, there are robots responsible for order picking across the entire warehouse, as well as dedicated robots assigned to individual clusters. Each robot in the system consists of a single 6-Degree-of-Freedom (DoF) unit mounted on an Unmanned Ground Vehicle (UGV).

As shown in the figure, the process begins with the modeling of the given warehouse. Accordingly, we develop two levels of modeling: one at the global level for the entire warehouse and another at the local level for individual clusters. One of these models is constructed using symbolic discrete controller synthesis methods, while the other is developed through metaheuristic approaches. Furthermore, metaheuristic techniques have been employed to solve the inverse kinematics challenges encountered in robotic arm systems. Finally, the synthesized controllers are integrated in the MATLAB environment and evaluated through experimental validation.

### 3.1. Overview

In our study, we address path optimization for warehouse robots within the scope of cluster order picking, adopting a hybrid modeling approach that combines symbolic control and metaheuristic algorithms. The warehouse considered in our study consists of multiple clusters, and each cluster is composed of shelves located along designated paths within the cluster. Within each cluster, specialized robots operate between the intra-cluster picking area and the shelves, performing item retrieval tasks. In addition, robots assigned to the entire warehouse operate between the cluster-level picking areas and the central warehouse picking area. All robots are equipped with a 6-DOF robotic manipulator mounted on a UGV. In this context, we investigate three distinct modeling approaches: (i) an inverse kinematics model for the 6-DOF manipulators to enable item picking operations; (ii) a metaheuristic-based method for path optimization; and (iii) a symbolic modeling approach, which is combined with the metaheuristic method to form a hybrid solution.

In the context of the warehouse cluster order picking system described above, we first address the inverse kinematics problem of the 6-DOF manipulators responsible for the picking task using metaheuristic approaches. The inverse kinematics problem is modeled through a BiLSTM-based deep learning method, utilizing the forward kinematic model defined by transformation matrices. The BiLSTM model is then optimized using the Taguchi method.

For cluster order picking, the path planning of warehouse robots is modeled using a symbolic discrete controller synthesis method. This is encoded as discrete events and modeled in the ReaX environment as parallel synchronous languages. Within this context, two types of modeling are considered: one for the robots operating within the general warehouse and another for the robots assigned to each individual cluster.

As in the symbolic model, both the robots operating within the entire warehouse and those specifically defined for each cluster are optimized using metaheuristic algorithms. Subsequently, based on the results derived from the experimental comparisons, the models to be used are selected, leading to the development of a hybrid approach.

### 3.2. Inverse Kinematics Model of 6-DoF Robotic Arms

To obtain the datasets for this study, the forward kinematic model of the manipulator is developed. The transformation matrices are derived by considering the coordinate transformation from the base frame to the manipulator’s end-effector. The homogeneous transformation matrix, as expressed in Equation (6), is obtained by sequentially multiplying the transformation matrices defined in Equations (3)–(5). In this formulation, $R_{k}$ and $T_{k}$ represent the rotation and translation matrices associated with the *k*-th joint, respectively.

(3) $$\begin{array}{cccc} R_{x} & =\left[\begin{array}{cccc} 1 & 0 & 0 & 0\\ 0 & \cos\alpha & -\sin\alpha & 0\\ 0 & \sin\alpha & \cos\alpha & 0\\ 0 & 0 & 0 & 1 \end{array}\right]\; & T_{x} & =\left[\begin{array}{cccc} 1 & 0 & 0 & a\\ 0 & 1 & 0 & 0\\ 0 & 0 & 1 & 0\\ 0 & 0 & 0 & 1 \end{array}\right] \end{array}$$

(4) $$\begin{array}{cccc} R_{y} & =\left[\begin{array}{cccc} \cos\beta & 0 & \sin\beta & 0\\ 0 & 1 & 0 & 0\\ -\sin\beta & 0 & \cos\beta & 0\\ 0 & 0 & 0 & 1 \end{array}\right]\; & T_{y} & =\left[\begin{array}{cccc} 1 & 0 & 0 & 0\\ 0 & 1 & 0 & b\\ 0 & 0 & 1 & 0\\ 0 & 0 & 0 & 1 \end{array}\right] \end{array}$$

(5) $$\begin{array}{cccc} R_{z} & =\left[\begin{array}{cccc} \cos\gamma & -\sin\gamma & 0 & 0\\ \sin\gamma & \cos\gamma & 0 & 0\\ 0 & 0 & 1 & 0\\ 0 & 0 & 0 & 1 \end{array}\right]\; & T_{z} & =\left[\begin{array}{cccc} 1 & 0 & 0 & 0\\ 0 & 1 & 0 & 0\\ 0 & 0 & 1 & c\\ 0 & 0 & 0 & 1 \end{array}\right] \end{array}$$

(6) $$T=\prod_{k=1}^{n}\left(R_{k}T_{k}\right)$$

**Figure 4:**
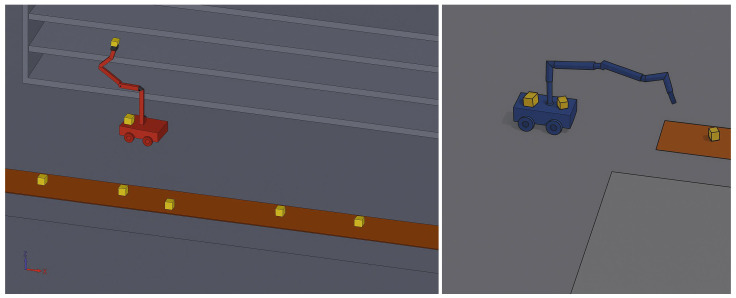
General view of the 3D model of the manipulator and the 6-DOF mounted UGV, i.e., the picker robot.

**Figure 5:**
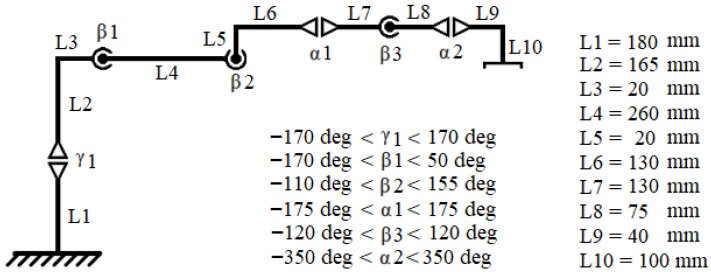
Kinematic scheme, link sizes, and limits of joint angles.

An overview of the manipulator’s 3D representation is presented in Figure 4. The kinematic scheme of the robot manipulator, along with the link dimensions and the rotational limits of the joint angles, is presented in Figure 5.

This work utilizes machine learning techniques to derive the inverse kinematic model of a robotic arm. Initially, an ANN model with two hidden layers was implemented to accurately model the inverse kinematics of the manipulator. In addition to the ANN, a Bidirectional Long Short-Term Memory (BiLSTM) network was also used. The BiLSTM architecture has the potential to capture complex temporal dependencies in the data. BiLSTM is a type of deep neural network capable of processing temporal sequences in both forward and backward directions. Similar to the ANN model, the hyperparameters of the BiLSTM network were optimized using the Taguchi method to ensure effective learning and improved predictive performance.

### 3.3. Symbolic Modeling for Cluster Order Picking

For path optimization in cluster order picking with warehouse robots, we apply the symbolic discrete controller synthesis method. This method is modeled using synchronous parallel programming languages for both central picking robots in the general warehouse and intra-cluster picking robots in a single cluster.

#### 3.3.1. PrinciplesUnderlying Our Approach

The principles of control theory for discrete event systems can be explained using two Mealy machines. Initially, consider two separate Mealy machines, denoted as A and B, where both machines have states 0 and 1. Let us assume that the transition between these states is carried out by a signal c. The desired system behavior is that both Mealy machines simultaneously be in the states 0 and 1. In this case, the desired system behavior can be achieved by the synchronous parallel composition of the Mealy machines A and B with a third Mealy machine, denoted as S, and the encapsulation of the signal c. In other words, the desired system behavior can always be guaranteed through the controllable variable c of the controller S.

In line with the control theory of discrete event systems, the models of central picking robots and intra-cluster picking robots, encoded in the ReaX environment for our safety and optimization algorithms, are detailed below.

#### 3.3.2. Central Warehouse Model

**Figure 6:**
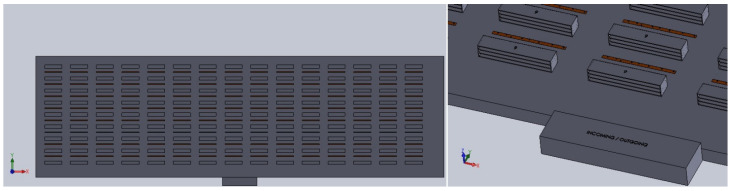
The layout of the warehouse, consisting of clusters, internal aisles, shelves, and central warehouse picking area.

In constructing the central warehouse model, all the intersections of the paths shown in Figure 6 are modeled as states. The paths between these nodes are associated with a controller, allowing transitions from one node to another via a controllable variable. The model is encoded as follows:

(7) $$x_{i}\overset{c_{ij}}{\to }x_{j},$$

where $x_{i}$ and $x_{j}$ represent the nodes between paths, where $i,j\in N_{w}$, and $N_{w}$ denotes the total number of nodes. $c_{ij}$ is the controllable input variable that ensures the transition from node $x_{i}$ to node $x_{j}$.

The equation for reaching the target destination from any given node is encoded as follows:

(8) $$x_{i}\overset{c_{iT}}{\to }x_{T},$$

where *T* is the target address.

The cost function we have formulated is structured in a cascading manner, similar to the branches and leaves of a tree, and is expressed using an ’if-then-else’ structure as follows:

(9) $$\Delta_{w}=\left\{\begin{array}{cc} \Delta_{i,j}, & \mathrm{if}\;x_{i,j}\\ \Delta_{i,k}, & \mathrm{if}\;x_{i,k}\\ \vdots & \\ \Delta_{i,n}, & \mathrm{if}\;x_{i,n}\\ \vdots & \\ \Delta_{n,i}, & \mathrm{if}\;x_{n,i}\\ \Delta_{n,j}, & \mathrm{if}\;x_{n,j}\\ \vdots & \\ \Delta_{n,k}, & \mathrm{if}\;x_{n,k} \end{array}\right.$$

#### 3.3.3. Intra-Cluster Model

**Figure 7:**
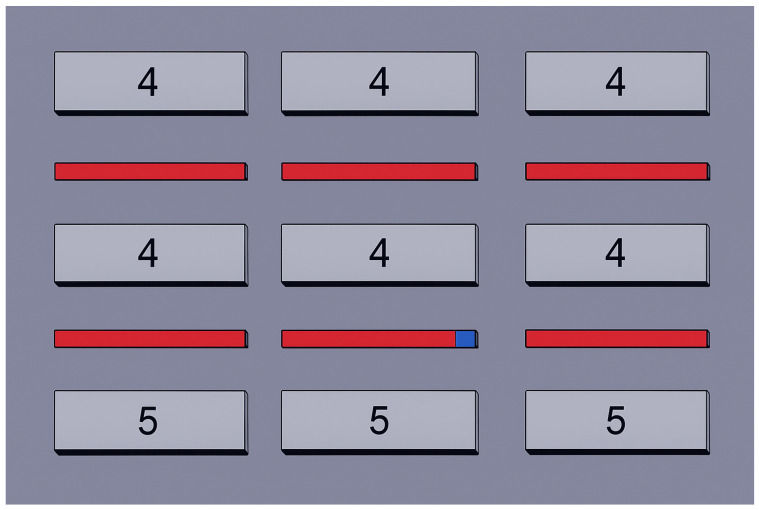
The layout of a single cluster, consisting of internal aisles, shelves (indicating cluster ID, as shown with the numbers 4 and 5), intra-cluster picking area (red-line), and the intra-picking and central picking robots (blue-box).

Similarly, for the intra-cluster picking robots in Figure 7, the transitions between the nodes within the cluster are encoded as follows:

(10) $$y_{i}\overset{c_{ij}}{\to }y_{j},$$

where $y_{i}$ represents the intra-cluster picking robots. The final location can be reached using the following equation:

(11) $$y_{i}\overset{c_{iT}}{\to }y_{T}.$$

The cost function for the intra-cluster robots is as follows:

(12) $$\Delta_{i}=\left\{\begin{array}{cc} \Delta_{i,j}, & \mathrm{if}\;y_{i,j}\\ \Delta_{i,k}, & \mathrm{if}\;y_{i,k}\\ \vdots & \\ \Delta_{i,n}, & \mathrm{if}\;y_{i,n}\\ \vdots & \\ \Delta_{n,i}, & \mathrm{if}\;y_{n,i}\\ \Delta_{n,j}, & \mathrm{if}\;y_{n,j}\\ \vdots & \\ \Delta_{n,k}, & \mathrm{if}\;y_{n,k} \end{array}\right.$$

#### 3.3.4. Global Collision Avoidance

To prevent collisions between all warehouse robots on the same paths between nodes, our mutual constraints on shared resources are expressed in the following equation:

(13) $$M=\underset{i,j\in N}{⋀}\left(x_{i}\wedge c_{ij}\right)\wedge \neg \left(\left(x_{j}\wedge c_{ji}\right)\vee \left(y_{j}\wedge c_{ji}\right)\right),$$

where:

- M: Global mutual exclusion condition for collision avoidance;
- $x_{i}$: Central robot presence at node *i*;
- $y_{j}$: Intra-cluster robot presence at node *j*;
- $c_{ij}$: Control inputs for transitions from *i* to *j*;
- ∧, ∨, ¬, and ${⋀}_{i,j\in N}$: Logical AND, OR, NOT, and a universal AND over all node pairs i,j in the set N.

#### 3.3.5. Global Control Objectives

The mutual exclusion constraints, which define strict rules, and the system states that fall under our safety objectives are evaluated through controllable variables using an invariant that always holds a value of 1. These are encoded as follows:

(14) $$S=M\wedge ⋀x_{i}\wedge ⋀y_{i}$$

The cost functions of the central warehouse and intra-cluster picker robots are cumulatively aggregated and minimized over a defined time window, as formulated below:

(15) $$O=f_{min}\left(\sum \Delta_{w}+\sum \Delta_{i}\right)$$

Finally, the controller obtained through the application of our safety and optimization synthesis algorithms to the model is integrated into the robotic system for path optimization.

### 3.4. MH-Based Path Planning for Autonomous Warehouse Robots

In our approach, metaheuristic methods are applied for path planning both at a global level and within single clusters. The results obtained are then hybridized with the symbolic discrete controller synthesis method. This section presents the proposed metaheuristic approach in detail.

In the field of MH methods, many algorithms have been developed in recent years. Various types of MH algorithms, such as human-based, evolutionary, physics-based, and nature-based, have been applied to a wide range of problems. Among these, swarm-based metaheuristic algorithms have become particularly popular. Recently developed and widely known MH algorithms are listed in Table 1.

In path planning, the aim is to calculate the shortest path that results in minimum energy consumption for the robots. To achieve this, the waypoints, defined as the junctions and the cells of the temporary storage line in this study, should first be identified. Incoming and outgoing package points are also defined as waypoints. The optimal paths are calculated based on the closest waypoint to each robot and the target waypoints. MH algorithms calculate the optimal paths in an iterative process before the robot begins its movement, while the SDCS method directs the robots during their navigation.

Advanced path planning methods developed for mobile robots help reduce wear and tear on the robots. Recent advancements in path planning technology have significantly increased the efficiency of unmanned robots across various fields. Mobile robot path planning involves determining the shortest and collision-free paths for autonomous movement. MH methods are widely used in both academic and industrial applications, involving single or multiple robots. To apply MH methods, a distance matrix is generated by calculating the distances between predefined waypoints. Using this matrix, MH algorithms attempt to find the optimal solution.

**Figure 8:**
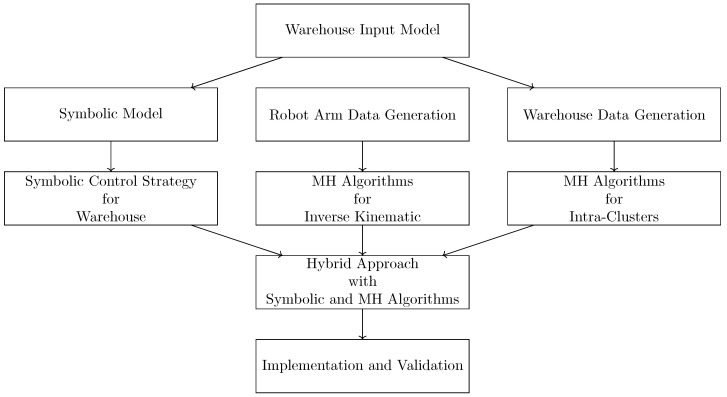
Data flow diagram of the proposed hybrid strategy combining symbolic discrete controller synthesis and metaheuristic optimization algorithms for warehouse cluster order picking.

The data flow of the proposed hybrid strategy is illustrated in Figure 8. A step-by-step representation of the study is provided in Algorithm 1. The proposed algorithm integrates symbolic control with metaheuristic techniques to optimize the path planning of robots deployed in warehouse environments.

**Algorithm 1** Hybrid Symbolic Control and Metaheuristic Path Optimization**Initialize:** Warehouse environment E, robot locations R, task locations T.**Set parameters:** Population size *P*, maximum iterations $I_{\max}$, mutation rate μ, crossover rate ρ.**Initialize symbolic controller:** $C_{\mathrm{sym}}$ that maps R to paths.**Generate initial population:** $P_{0}=\left\{r_{1},r_{2},\ldots ,r_{P}\right\}$ using $C_{\mathrm{sym}}$.**Evaluate initial population:** $J(r_{i})$ for each path $r_{i}\in P_{0}$.**for** each metaheuristic algorithm $A_{i}$ **do**    **Initialize:** Population $P_{i}=\left\{r_{1},\ldots ,r_{P}\right\}$.

    **Evaluate:** $J(r_{i})$ for each path $r_{i}\in P_{i}$.
    **for** each iteration t=1 to $I_{\max}$ **do**        **Selection:** Choose parents based on fitness: $P_{i}^{\mathrm{parents}}$.
        **Crossover:** Apply crossover to parents to produce offspring: $P_{i}^{\mathrm{offspring}}$.
        **Mutation:** Apply mutation to offspring with rate μ.
        **Evaluate offspring:** $J(r_{j})$ for each offspring $r_{j}$.
        **Select next generation:** $P_{i}^{\mathrm{next}}=\mathrm{Select}\left(P_{i}^{\mathrm{parents}}\cup P_{i}^{\mathrm{offspring}}\right)$.
    **end for**
**end for**

**Select best solution:**

$$s_{\mathrm{best}}=\arg\min_{s\in P_{i}}J\left(s\right)$$

**Evaluate final path:** $J_{f}\left(s_{\mathrm{best}}\right)=\sum_{t\in T}{∥s_{\mathrm{best}}\left(t\right)-T\left(t\right)∥}^{2}$.
**Output final paths:** $s_{\mathrm{best}}$.


## 4. Experimental Evaluation

This study includes evaluations in three main areas: the inverse kinematics problem, path optimization for warehouse robots using metaheuristic algorithms, and symbolic controller synthesis for path planning. First, a dataset was generated for the application of metaheuristic algorithms. In parallel, a symbolic model was developed to synthesize a controller. All control structures, including those derived from the symbolic controller, were implemented in the MATLAB environment. Comparative evaluations were then conducted and reported accordingly.

The experiments involved grid-based warehouse layouts of 10,000, 20,000, and 30,000 $m^{2}$ with 30, 50, and 70 robots, with the initial positions shown in Figure 6. A dataset of 1.7 million samples was generated by simulating a 6-DOF robot manipulator’s joint angles (−50 to +50 degrees) and corresponding end-effector positions to train the inverse kinematics model. Forward kinematics was used to calculate motion parameters for each setup. The dataset was validated through MATLAB Simulink and physical experiments.

First, our evaluation related to the inverse kinematics problem is presented as follows. Obtaining the optimal settings for ANN parameters, such as neuron number of 1st hidden layer, neuron number of 2nd hidden layer, and percentage of the training data, has a time-consuming trial and error process. To obtain the most successful ANN model, the Taguchi L27 orthogonal array was utilized in this study. Table 2 summarizes the coefficient of determination $R^{2}$ results of the ANN models. The performance of deep learning models depends on the tuning of hyperparameters. Achieving optimal settings for hyperparameters, such as the training dataset, learning rate, number of epochs, batch size, and optimization algorithm, requires expertise and extensive trial and error. To obtain the most effective deep learning model, the Taguchi L27 orthogonal array was utilized in this study. Table 3 summarizes $R^{2}$ values of the deep learning models.

The success of a model is evaluated using statistical criteria such as the $R^{2}$ value and Mean Squared Error (MSE). According to Table 2, the results are relatively close to each other. However, the highest $R^{2}$ value was obtained when neuron number of 1st hidden layer, neuron number of 2nd hidden layer, and percentage of the training data are selected as 10, 20, and 90, respectively. As shown in Table 3, the results are relatively close to each other. However, the highest $R^{2}$ value was achieved when the optimizer algorithm, learning rate, and batch size were set to Adam, 0.01, and 5000, respectively. Therefore, the BiLSTM model was implemented using these optimized parameter values.

A constant learning rate was selected for training to maintain stability and simplicity. While adaptive or piecewise schedules can accelerate convergence, our preliminary tests showed that a fixed rate provided consistent and reliable results for this application.

Following the evaluation of the inverse kinematics problem, the path optimization results for both central warehouse robots and intra-cluster robots, obtained using metaheuristic algorithms and symbolic control, are presented below.

Picker-carrier robots were assigned to retrieve outgoing packages from the temporary storage line, where the packages had been placed by the cluster-picker robots. They were also responsible for placing incoming packages onto the line, enabling the cluster-picker robots to subsequently store them on the racks. Cluster-picker robots were assigned to retrieve incoming packages from the temporary storage line, where the packages had been placed by the picker-carrier robots, and to store them on the racks. They were also responsible for retrieving outgoing packages from the racks and placing them onto the line. Table 4 presents the total path lengths traveled by both intra-cluster and cluster-picker robots when various iteration numbers and populations sizes/k steps were tested.

According to Table 4, the SDCS algorithm with 5 k-step produced the shortest path, measuring 1257 m for intra-cluster length. Additionally, the PO algorithm emerged as the most effective MH algorithm, consistently calculating the same 1257 m path across all tested population sizes. As shown in Table 4, varying the population size did not lead to improved results. Since the PO algorithm provides the optimal solution in every case and has lower implementation complexity compared to the SDCS algorithm, it is preferred for the path planning of the picker-carrier robots. On the other hand, the SDCS algorithm with 5 k-step produced the shortest path, measuring 6117 m for cluster picker length. Additionally, the PO algorithm was the most successful MH algorithm, yielding a 6134 m path when the population size was set to 100. Varying the population size did not lead to performance improvements. However, the MH algorithms did not yield the optimal solution in any case; therefore, they cannot be used as substitutes for the SDCS algorithm in the path planning of cluster-picker robots.

**Figure 9:**
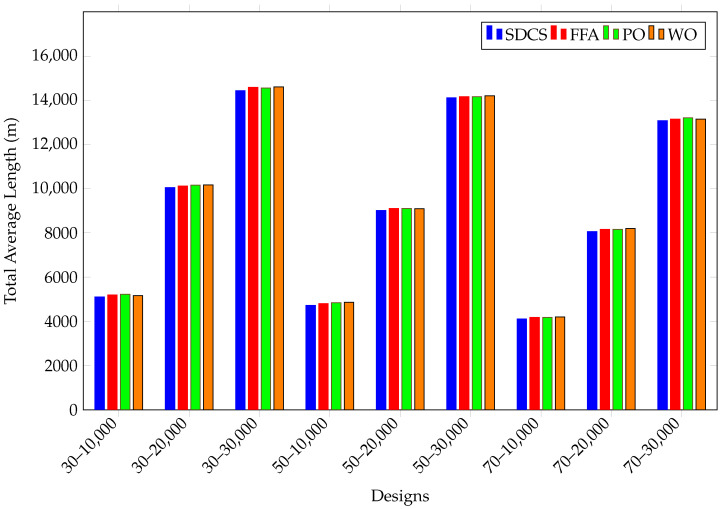
The results of the tests conducted using various numbers of robots in warehouses of different sizes.

The results of all designs are shown in Figure 9. The designs include trials conducted in warehouses of 10,000, 20,000, and 30,000 square meters, with 30, 50, and 70 robots. When the results in the figure are examined, it can be observed that as the warehouse size increases, the total distance covered by the robots also increases. However, as the number of robots increases, the total distance covered by each robot decreases. The best results were obtained using the SDCS algorithm in all cases, although the MH algorithms produced results close to those of SDCS. None of the MH algorithms showed a significant advantage over the others. Increasing the number of robots may reduce operating costs, but it will certainly increase investment costs.

Less conventional MH algorithms (WO, PO, FFA) were chosen for their strong exploration–exploitation balance and success in complex, multi-modal problems. Preliminary tests with classical methods (PSO, ACO) showed comparable or better performance when tuned via Taguchi. This ensures a solid benchmark against the symbolic controller.

In the literature, numerous studies have proposed MH-based solutions for warehouse optimization. Ref. [31] introduced an innovative methodology that integrates machine learning with genetic algorithms to address the warehouse optimization problem. Specifically, a non-linear predictive model based on machine learning is employed to estimate the picking time for batches of orders, utilizing both quantitative and spatial characteristics of the batches, as well as indicators related to picker learning and fatigue. These predictions are subsequently used to inform a genetic algorithm designed to optimize the assignment of upcoming order batches to individual pickers. Ref. [32] conducted a comparative analysis of genetic algorithms and simulated annealing techniques using actual operational data. Their objective was to minimize picker travel distances, optimize routing processes, and enhance overall operational efficiency within order-picking systems in the medical textile industry. Ref. [33] investigated an alternative configuration for warehouse systems involving multiple pickers and depots within manually operated picker-to-parts environments. To address the complexity of the problem, the authors proposed a novel bi-objective mixed-integer linear programming model suitable for small-scale instances, along with a metaheuristic approach termed Dependent Harmony Search (DHS) for larger problem sizes. The effectiveness of the DHS algorithm was evaluated by benchmarking its results against the optimal solutions obtained from the MILP formulation.

When the results of these and similar studies are examined, it becomes evident that MH algorithms yield optimal or near-optimal outcomes. Although the SDCS method requires extensive pre-implementation processes and a simulation environment, it offers a single, optimal solution. In this context, it is anticipated that the approaches presented in the literature would achieve optimal results if the robots were modeled using Simscape and simulated in the Simulink environment, incorporating the SDCS method for path planning.

## 5. Conclusions and Future Work

In this study, the cluster order picking problem for warehouse robots is addressed. A systematic modeling approach is proposed, integrating hybrid symbolic control and metaheuristic techniques for path planning optimization. The resulting controller is employed as a cluster manager, and experimental evaluations are conducted to assess its performance. The findings validate the effectiveness of our hybrid approach, demonstrating superior performance relative to previously reported techniques.

The DCS method offers several advantages, including the provision of exact and optimal solutions without the need for parameter optimization during pre-processing, which is typically required in Metaheuristic (MH) algorithms. Furthermore, the DCS method demonstrates adaptability to dynamic environments, such as waypoint updates in path planning tasks. The proposed framework seeks to establish systematic principles for modeling the order-picking problem and to facilitate the integration of the DCS controller within a simulation environment. One notable limitation of the SDCS approach is the necessity for researchers to utilize multiple software platforms, such as ReaX and Simulink, in tandem. Despite this drawback, the SDCS method remains a preferable alternative to conventional approaches in control and path planning applications, due to its capacity to deliver accurate and optimal performance outcomes.

One of the biggest limitations of our approach is scalability. The size of the warehouse area or the increase in the number of clusters does not pose a problem in terms of execution time. However, since the robots are modeled as states, a significant increase in the number of states greatly increases the synthesis time of the controller. That said, this situation only arises when the number of robots in a cluster increases excessively. In realistic scenarios, a significant increase in the number of robots is typically accompanied by an increase in the number of clusters, which means that the number of robots within a single cluster does not grow excessively. This allows us to handle the problem using smaller DCS problems. As a note, the synthesis process is performed only once; once the controller is synthesized, the system responds dynamically in real time.

The approach presented in this study can be seamlessly integrated into similar systems in future work. Its applicability extends to path planning in unmanned ground vehicles and aerial robots, demonstrating significant suitability for such domains. While this study focuses on path planning optimization, the proposed methodology can also be adapted to address performance criteria such as energy efficiency and time optimization in various applications. Furthermore, multi-criteria objectives can be incorporated, enabling the development of diverse control strategies. The metaheuristic optimization algorithms employed within the hybrid system can be enriched and customized for specific requirements. Lastly, the control algorithms discussed herein can be safely integrated into safety-critical systems, where reliability is paramount, and can be tested with alternative controller synthesis methods.

## Figures and Tables

**Table 1:** Metaheuristic algorithms considered in this study, along with their types and years of development.

Algorithm	Year	Type
Grey Wolf Optimizer	2014	Swarm-based
Ant Lion Optimizer	2015	Nature-based
Dragonfly Algorithm	2016	Swarm-based
Grasshopper Optimization Algorithm	2017	Swarm-based
Owl Search Algorithm	2018	Nature-based
Butterfly Optimization Algorithm	2019	Nature-based
Black Widow Optimization Algorithm	2020	Human-based
African Vultures Optimization Algorithm	2021	Nature-based
White Shark Optimizer	2022	Nature-based
Flying Fox Optimization Algorithm	2023	Nature-based
Puma Optimizer	2024	Nature-based
Walrus Optimizer	2024	Nature-based

**Table 2:** $R^{2}$ values of the artificial neural network models (HL: Hidden Layer; T: Percentage of Training Data).

Params.	HL1 = 10			HL1 = 20			HL1 = 30		
	T = 70	T = 80	T = 90	T = 70	T = 80	T = 90	T = 70	T = 80	T = 90
HL2 = 10	0.910	0.921	0.924	0.923	0.930	0.906	0.923	0.930	0.906
HL2 = 20	0.924	0.929	0.932	0.923	0.925	0.929	0.923	0.925	0.929
HL2 = 30	0.928	0.924	0.931	0.923	0.930	0.915	0.925	0.927	0.915

**Table 3:** $R^{2}$ values for various optimizers, learning rates (LR), and batch sizes (BS).

Params.		Optimizer								
		Adam			Nadam			SGD		
	LR	0.1	0.01	0.001	0.1	0.01	0.001	0.1	0.01	0.001
BS										
1000		0.978	0.964	0.960	0.931	0.962	0.975	0.944	0.965	0.936
3000		0.983	0.976	0.934	0.979	0.958	0.937	0.973	0.938	0.956
5000		0.957	0.985	0.955	0.935	0.938	0.965	0.939	0.951	0.932

**Table 4:** Comparison of algorithms with different population sizes/k steps on total path length for both intra-cluster and cluster picker robots across various iteration numbers.

Algorithm	Population Size/k Steps	Intra-Cluster Length (m)			Cluster Length (m)		
		Iter. 100	Iter. 200	Iter. 300	Iter. 100	Iter. 200	Iter. 300
SDCS	1	1286 (no iter.)			6138 (no iter.)		
	3	1278 (no iter.)			6126 (no iter.)		
	5	1257 (no iter.)			6117 (no iter.)		
FFA	100	1296	1278	1278	6167	6145	6145
	200	1257	1257	1286	6232	6134	6148
	300	1278	1278	1278	6167	6245	6245
PO	100	1257	1257	1257	6134	6232	6232
	200	1257	1257	1286	6134	6145	6138
	300	1257	1278	1278	6167	6213	6213
WO	100	1278	1278	1278	6245	6167	6167
	200	1278	1296	1296	6213	6232	6148
	300	1257	1278	1286	6245	6245	6245

## Data Availability

Data are contained within the article.
